# 4-1BB Signaling Boosts the Anti-Tumor Activity of CD28-Incorporated 2^nd^ Generation Chimeric Antigen Receptor-Modified T Cells

**DOI:** 10.3389/fimmu.2020.539654

**Published:** 2020-11-13

**Authors:** Qiang Dai, Ping Han, Xinyue Qi, Fanlin Li, Min Li, Lilv Fan, Huihui Zhang, Xiaoqing Zhang, Xuanming Yang

**Affiliations:** ^1^Sheng Yushou Center of Cell Biology and Immunology, School of Life Sciences and Biotechnology, Shanghai Jiao Tong University, Shanghai, China; ^2^Joint International Research Laboratory of Metabolic & Developmental Sciences, Shanghai Jiao Tong University, Shanghai, China; ^3^Key Laboratory of Systems Biomedicine (Ministry of Education), Shanghai Center for Systems Biomedicine, Shanghai Jiao Tong University, Shanghai, China

**Keywords:** 4-1BB, chimeric antigen receptor-modified T, lymphoma, immunotherapy, CD20

## Abstract

While chimeric antigen receptor-modified T (CAR-T) cells have shown great success for the treatment of B cell leukemia, their efficacy appears to be compromised in B cell derived lymphoma and solid tumors. Optimization of the CAR design to improve persistence and cytotoxicity is a focus of the current CAR-T study. Herein, we established a novel CAR structure by adding a full length 4-1BB co-stimulatory receptor to a 28Z-based second generation CAR that targets CD20. Our data indicated that this new 2028Z-4-1BB CAR-T cell showed improved proliferation and cytotoxic ability. To further understand the mechanism of action, we found that constitutive 4-1BB sensing significantly reduced the apoptosis of CAR-T cells, enhanced proliferation, and increased NF-κB pathway activation. Consistent with the enhanced proliferation and cytotoxicity *in vitro*, this new structure of CAR-T cells exhibited robust persistence and anti-tumor activity in a mouse xenograft lymphoma model. This work provides evidence for a new strategy to optimize the function of CAR-T against lymphoma.

## Introduction

B cell-derived malignancies represent a diverse population of diseases including various types of leukemia and lymphoma (1, 2). Rituximab combined with chemotherapy is a first line treatment for B cell-derived lymphoma (3, 4). Despite that this treatment is effective for a large proportion of patients, approximately 30–60% of patients are not responsive to this treatment, or undergo relapse after initial response (5). Hence, novel treatments are urgently needed for these refractory and relapsing lymphoma patients. Chimeric antigen receptor (CAR) T cell therapy is one of the most revolutionary breakthroughs for immunotherapy (6). It has achieved ~80% complete remission rate in treating CD19^+^ relapsed/refractory aggressive B cell leukemia (7). However, the complete remission rate significantly decreased to approximately 30–60% when the same treatment was applied to B cell lymphoma (8). Understanding how leukemia and lymphoma differentially affect the function of CAR-T cells, and the development of new CAR-T strategy, is important to improve the therapeutic outcome in lymphoma treatment.

The molecular design of CARs determines the functional activity of CAR-T cells *in vitro* and *in vivo*. Current clinically applied CARs consist of an extracellular antigen-binding domain, a hinge and transmembrane domain, co-stimulatory domain, and a CD3ζ activation domain (9). Optimization of the CAR design to improve its persistence and cytotoxicity is a focus of current CAR-T studies (10–12). Various approaches have been reported to achieve these goals. For instance, some strategies have incorporated additional components into the current CAR design, including IL-7, CCL19, scFv-PD-1, CD40L, membrane-anchored IL-15, and BITE-EGFR (13–17). These strategies not only enhance the intrinsic function of CAR-T cells, but also allows them to acquire novel immune regulatory or direct anti-tumor abilities. Other strategies have focused on engineering the essential components of CAR design (11). Using low affinity antigen-binding domains in the CAR design has shown improved anti-tumor activity in different tumor models (18). Also, optimization of the linker attached to the hinge and transmembrane domain has facilitated improved persistence and reduced CAR-T cell side effects (19).

In addition to the TCR signal, co-stimulation pathways provide critical signals for T cell activation and proliferation (20). Multiple co-stimulatory receptors are inducibly expressed during T cell activation and differentiation (20). 4-1BB (CD137) is a critical co-stimulatory receptor primarily expressed by activated CD8^+^ T cells, and is a member of the tumor necrosis factor receptor super family (21). Following engagement by natural ligand 4-1BBL, 4-1BB transduces the activation signal of NF-κB, which induces preferential expansion and activation of CD8^+^ compared to CD4^+^ T cells (22). By utilizing this mechanism, agonistic antibodies to 4-1BB have shown promising anti-tumor efficacy in preclinical models (23). 4-1BB and CD28 intracellular domains are widely coupled with the CD3ζ intracellular domain to deliver CAR activation signals. Although several studies have demonstrated that the CD28 co-stimulatory domain confers more rapid expansion, it is also associated with poor persistence in CAR-T cells (24–26). Conversely, 4-1BB-containing CAR-T cells have prolonged persistence (27, 28). Hence, in the current CAR design, the 4-1BB signal is integrated into the CAR design. During the natural T cell activation course, the 4-1BB signal is separated to the TCR signal. Whether 28Z based CAR-T cells could benefit from an additional separated 4-1BB signal has not been investigated.

Herein, we established a novel CAR structure by adding a constitutive 4-1BB co-stimulatory receptor to a 28Z-based second generation CAR structure targeting CD20. Compared with 2028Z CAR-T cells, 2028Z-4-1BB CAR-T cells showed multiple advantages in *in vitro* culture including: enhanced proliferation and tumor killing capacity, as well as reduced apoptosis and exhaustion. Furthermore, 2028Z-4-1BB CAR-T cells showed improved persistence and tumor control *in vivo* in a lymphoma xenograft mouse model. This work provides evidence for a new strategy to optimize the function of CAR-T cells against lymphoma.

## Materials and Methods

### Cell Lines

The Lenti-X 293 cell line was purchased from Clontech (Mountain View, CA). The Raji cell line and Daudi cell line were purchased from Chinese Academy of Sciences (Shanghai, China). The NALM-6 cell line was purchased from American Tissue Culture Collection (ATCC, Manassas, VA). NALM-6 was infected with lentivirus expressing human CD20 and subcloned by limited dilution to generate NALM-6-hCD20. Lenti-X 293 was cultured in DMEM. Raji, NALM-6 and NALM-6-hCD20 were maintained in RPMI-1640. All cell culture mediums were supplemented with 10% heat-inactivated fetal bovine serum (Gibco), 2 mmol/L L-glutamine, 100 units/ml penicillin, and 100 μg/ml streptomycin.

### CAR Design and Lentivirus Production

CAR antigen-targeting regions, scFv, were derived from Rituximab. 2028Z CAR consisted of the scFv linked to intracellular signaling domain containing CD28 and CD3ζ by CD8α hinge and CD28 transmembrane domain. 4-1BB was linked to CD3ζ, by P2A peptide, to generate 2028Z-4-1BB.

2028Z and 2028Z-4-1BB CAR coding DNA were cloned into a modified pCDH-EF1-MSC vector backbone (Palo Alto, CA, USA) to generate a lentiviral transfer vector. The lentivirus has been produced by Lenti-X 293 cells according to a previously described protocol (29).

### CAR-T Cell Manufacture

Peripheral blood mononuclear cells (PBMCs) derived from cord blood were provided by Shanghai Longyao Biotechnology Co., Ltd. (Shanghai, China) and were isolated by Ficoll-Paque density-gradient centrifugation. Total T cells were purified with an EasySep™ Human T Cell Isolation kit (Stemcell). Purified T cells were seeded into a 96-well plate and stimulated with plate-bound anti-CD3 (0.25 μg/ml) and anti-CD28 (1 μg/ml) antibodies for 72 h. Activated T cells were then transduced with lentivirus encoding 2028Z CAR or 2028Z-4-1BB CAR at a multiplicity of infection (MOI) of 10. During *in vitro* expansion, CAR-T cells were stimulated weekly by irradiated Raji cells. CAR-T cells were cultured in RPMI-1640 medium with 200 IU/ml IL-2 (Beijing Sihuan Biopharmaceutical Co., Ltd.), and 4 ng/ml IL-21 (#571208 Biolegend).

### *In Vitro* Killing Ability Assay

A total of 1 × 10^5^ CAR-T cells were incubated with NALM-6, NALM-6-hCD20 or Raji cells at different effector:target (E:T) ratios of 1:0.5, 1:1, 1:2 in 96-well plate. After plating (12 and 24 h) cells were harvested and analyzed by flow cytometry. Anti-CD3 (#317306, Biolegend) and anti-CD19 (#302212, Biolegend) antibodies were used to distinguish CAR-T and tumor cells, respectively.

### Cytokine Release Assay

A total of 1×10^5^ CAR-T cells were incubated with Raji cells at E:T ratios of 1:0.5, 1:1, 1:2 in 96-well plates for 12 h. The supernatant was harvested for detecting IFN-γ, TNF-α and IL-2 using a Cytometric Bead Array (CBA) kit (BD Biosciences) according to the manufacturer’s protocol.

### *In Vivo* Anti-Tumor Activity of CAR-T Cells

Female NOD/SCID/γ^−/−^ (NSG) mice were purchased from the Shanghai Model Organisms Center, Inc. (Shanghai, China). All mice were maintained under specific pathogen-free conditions. Animal care and use, biosecurity procedures and protocols were in accordance with institutional and NIH protocols and guidelines (NIH guidelines for research involving recombinant or synthetic nucleic acid molecules, April 2019, https://osp.od.nih.gov). All experiments and biosecurity procedures were approved by the Animal Care and Use Committee of Shanghai Jiao Tong University.

Mice were injected intravenously (i.v.) with 5×10^5^ Raji cells. One week after tumor inoculation, mice were randomly grouped and treated with PBS or 1×10^7^ 20208Z or 2028Z-4-1BB CAR-T cells. One week after injection with CAR-T cells, the percentage of CAR-T cells and Raji cells in the peripheral blood were assessed by flow cytometry. Nine days after CAR-T cell injection, mice were sacrificed, and the tumor burden and CAR-T cell persistence in bone marrow and spleen was analyzed by flow cytometry.

### Flow Cytometry

Single cell suspensions of cells were incubated with anti-CD16/32 (anti-FcγRII/III, clone 2.4G2) for 10 min and then subsequently stained with conjugated Abs. The antibodies used are as follows: anti-human CD45RO-APC-A700 (UCHL1), anti-human CD45RA-PB450 (HI100), anti-human CD62L-FITC (DREG-56), anti-human CD4-PB450 (OKT4), anti-human CD8α-APC-A750 (HIT8a), anti-human CD3-FITC (OKT3), anti-mouse CD45-PB450 (30-F11), and anti-human 4-1BB ligand PE (5F4) were purchased from Biolegend; anti-human PD-1-APC (eBioJ105), anti-human TIM-3-PE (F35-2E2), anti-human LAG-3-FITC (3DS223H), anti-human CD3-FITC (OKT3), anti-human CD45-APC (HI30), and anti-human CD19-FITC (HIB19) were purchased from eBioscience; anti-human CCR-7-APC (552176) was purchased from BD Bioscience; goat anti-mouse IgG and F(ab’)2- FITC were purchased from Jackson Immuno Research. Samples were analyzed on a Cytoflex (Beckman Coulter), and data were analyzed with FlowJo software (TreeStar, Inc.).

### Western Blot Analysis

2028Z or 2028Z-4-1BB CAR-T cells were stimulated with anti-4-1BB (1 μg/ml) antibody for 15 min, harvested, lysed, and separated by SDS-PAGE. Immune blotting was carried out with the following antibodies: anti-NF-κB p65 (1:1000, #8242, CST), anti-GAPDH (1:2000, #5174, CST), anti-p-NF-κB p65 (S536) (1:1000, #3033, CST), and IRDye^@^ 800CW goat anti-rabbit IgG secondary antibody (1:5000, 926-32211, LI-COR). All images were acquired using the Western Blot Detection System (LI-COR & Odyssey CLx).

### Quantitative, Reverse Transcription PCR (RT-qPCR)

A total of 1,000 ng of RNA was reverse transcribed to complementary DNA (cDNA) using the ReverTra Ace -α- (TOYOBO CO., LTD.) according to manufacturer’s instructions. RT-qPCR was performed using the ChamQ Universal SYBR qPCR Master Mix (Vazyme Biotech Co.,Ltd). Standard 2^-ΔΔCt^ quantification of cDNA amplification was performed with *Gapdh* as the reference gene. The following primer sequences were used (5′ ➜ 3′):

*Gapdh* forward: GGAGCGAGATCCCTCCAAAAT;
*Gapdh* reverse: GGCTGTTGTCATACTTCTCATGG;
Exogenous 4-1BB forward: CAGCCACCAAGGACACTACGA;
Exogenous 4-1BB reverse: AAGTTGAGGACCAGCAACAGAGT;
Endogenous 4-1BB forward: CGTTGCTCTTCCTGCTGTTCTTC;
Endogenous 4-1BB reverse: TCACAGTTCACATCCTCCTTCTTCT;
*Bcl2* forward: GGTGGGGTCATGTGTGTGG;
*Bc-2* reverse: CGGTTCAGGTACTCAGTCATCC.
The amplification program applied for qPCR was as follows:
Step 1, 95°C for 30 s
Step 2, 95°C for 10 s
Step 3, 60°C for 30 s
Step 4, 40 cycles of 95°C for 10 s

### Statistical Analysis

Statistical analyses were performed using GraphPad Prism version 6.0. Significance of the *in vitro* assay was determined by a two-sided Student’s unpaired t-test. A two-sided log rank test was applied to assess mouse survival. Where indicated, ∗ P < 0.05, ∗∗ P < 0.01, and ∗∗∗ P < 0.001 were considered statistically significant.

## Results

### Design and Generation of 2028Z-4-1BB CAR-T Cells

**Figure 1:**
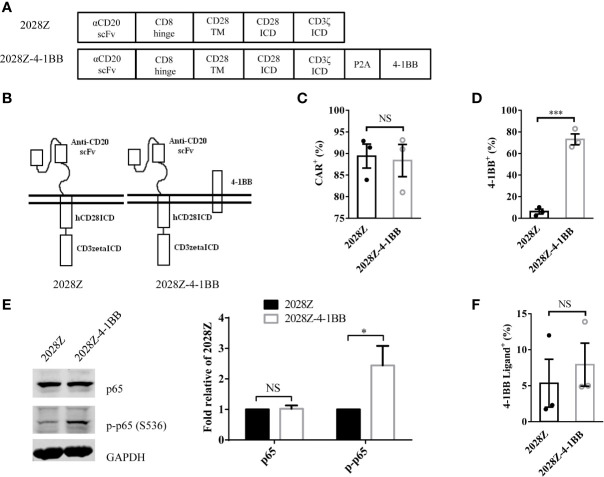
Characterization of 2028Z-4-1BB CAR-T cells. **(A, B)** A schematic diagram of the CD20 targeting CAR constructs used in this study. An anti-human CD20 scFv was linked to CD28 and CD3ζ to generate a 2028Z construct. 4-1BB was linked to CD3ζ *via* a P2A sequence. **(C, D)** Flow cytometry analysis of CAR **(C)** and 4-1BB **(D)** expression on 2028Z and 2028Z-4-1BB CAR-T cells. **(E)** Western blot analysis of phosphorylated p65 in 2028Z and 2028Z-4-1BB CAR-T cells. GAPDH was used as a loading control. **(F)** Flow cytometry analysis of 4-1BB Ligand expression on 2028Z and 2028Z-4-1BB CAR-T cells. Representative results from one from three **(C, D)** and two **(E)** repeated experiments are shown. *P < 0.05, ***P < 0.001; NS: Not Significant.

Following integration of the 4-1BB intracellular domain, the CAR-T cells exhibited enhanced persistence *in vivo* compared with the CD28 intracellular domain (24–27). To determine whether separated 4-1BB signaling could provide enhanced persistence to CAR-T cells, we first constructed two CD20 targeting CARs, one referred to as 2028Z, and the other as 2028Z-4-1BB, in which an additional 4-1BB co-stimulatory molecule was linked to CD3ζ by a cleavable P2A peptide (GSGATNFSLLKQAGDVEENPGP) (**Figures 1A, B**). The 2A-based multicistronic protein expression is mediated by ribosome skipping (30). This fusion DNA is transcribed to an intact mRNA through the classical transcription machinery. During subsequent translation, 2028Z polypeptides break from 4-1BB between glycine and proline in the 2A region. The cleavage process occurs inside the ribosome during protein synthesis and does not affect the translation of subsequent 4-1BB protein. This process resulted in a 2028Z CAR with extra 21 amino acid residues at the C-terminal, and an intact 4-1BB with one single proline at the N-terminal. To evaluate whether the additional 2A-linked 4-1BB affects the expression of CAR on the cell surface, we infected primary human T cells from three donors with these two lentiviruses. The expressional levels of the CARs were comparable, suggesting that the additional 4-1BB did not affect the expression or localization of the CAR (**Figure 1C**). To further determine whether 2A-linked 4-1BB co-stimulatory receptor was properly processed, we analyzed its surface expression by flow cytometry. As expected, most 2028Z-4-1BB CAR-T cells expressed 4-1BB on the surface, while only a small proportion of 2028Z CAR-T cells expressed 4-1BB (**Supplementary Figure 1** and **Figure 1D**). To determine whether exogenous 4-1BB expression could enhance endogenous 4-1BB expression in 2028Z-4-1BB CAR-T cells, we compared the mRNA expression of exogenous or endogenous 4-1BB by quantitative PCR using differential probes. We found that exogenous 4-1BB mRNA was significantly increased in 2028Z-4-1BB CAR-T cells compared to 2028Z CAR-T cells. Meanwhile, the expression of endogenous 4-1BB mRNA was similarly low in both CAR-T cells. These results suggest that high expression of 4-1BB in 2028Z-4-1BB CAR-T cells primarily induced by exogenous transgene expression (**Supplementary Figure 2**).

Next, since, 4-1BB can trigger the activation of the NF-κB pathway (21), to assess whether 2A-linked 4-1BB was functional in 2028Z-4-1BB CAR-T cells, we compared the phosphorylation of p65 in 2028Z-4-1BB and 2028Z CAR-T cells. Results show that phospho-p65 in 2028Z-4-1BB CAR-T cells was significantly elevated (**Figure 1E**). Furthermore, we observed that both CAR-T cells (**Figure 1F** and **Supplementary Figure 3A**), as well as B cell leukemia and lymphoma cells, Raji, Nalm-6, and Daudi (**Supplementary Figure 3B**), expressed 4-1BB ligand, which provided a source of activation signaling to 4-1BB. Taken together, the separately expressed 4-1BB in CAR-T cells is functional and activation of the 4-1BB pathway may provide survival and activation benefits for CAR-T cells.

### 4-1BB Co-Stimulation Signal Alters 2028Z-4-1BB CAR-T Cells Differentiation

**Figure 2:**
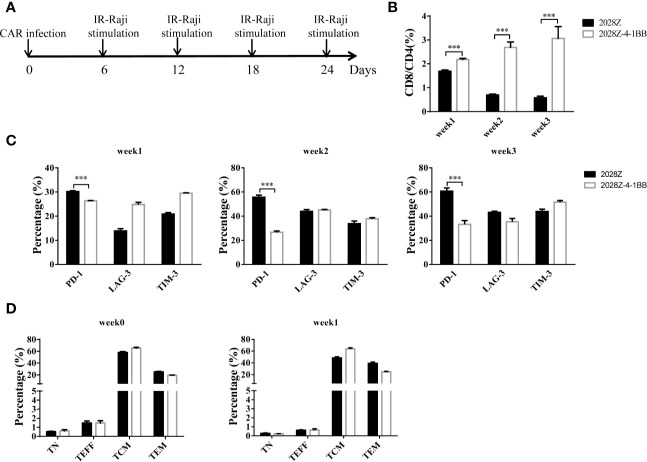
4-1BB signal alters 2028Z-4-1BB CAR-T cell differentiation. **(A)** A schematic diagram of the long-term CAR-T expansion assay. T cells were infected with the indicated CAR lentivirus and stimulated with irradiated Raji cells every six days. Total cell number was recorded and CAR^+^ cell percentage was analyzed by flow cytometry. **(B)** The CD4^+^ and CD8^+^ CAR-T cell percentage in the long-term culture was analyzed by flow cytometry. **(C)** The cell surface expression of exhaustion markers PD-1, TIM-3, and LAG-3 was measured in CAR-T cells after stimulation with irradiated Raji cells at the indicated time point. **(D)** Relative proportion of naive (CD45RA^+^CD45RO^-^CCR7^+^, TN), central memory (CD45RA^-^CD45RO^+^CCR7^+^, TCM), effector memory (CD45RA^-^CD45RO^+^CCR7^-^, TEM), and effector (CD45RA^+^CD45RO^-^CCR7^-^, TEFF) T cells in 2028Z and 2028Z-4-1BB CAR-T cells at the indicated time point. Representative results from one from three **(B, D)** repeated experiments are shown. ***P < 0.001.

The 4-1BB signal has been reported to enhance CD8^+^ T cell survival and promote memory CD8^+^ T cell development (22). To mimic the long-term exposure of CAR-T cells *in vivo*, we developed an *in vitro* repetitive antigen stimulation protocol (**Figure 2A**). We then analyzed whether additional 4-1BB signal could affect the differentiation of CAR-T cells, focusing on CD8^+^ and CD4^+^ cell differentiation, memory status, and exhaustion markers during the *in vitro* culture period. Interestingly, there was a slight increase in CD8 expression on 2028Z-4-1BB CAR-T cells compared to 2028Z CAR-T cells after the first week of long-term culturing. This difference consistently increased over the second and third week of long-term culture, suggesting that the 4-1BB signal predominantly affected the proliferation or survival of CD8^+^ CAR-T cells over CD4^+^ CAR-T cells (**Supplementary Figure 4** and **Figure 2B**). These results support the natural role of 4-1BB in unmodified T cells. To test whether 4-1BB signaling could affect the exhaustion status of CAR-T cells, we examined the exhaustion-related cell surface markers PD-1, TIM-3, and LAG- and observed that the expression of PD-1 was significantly lower in 2028Z-4-1BB CAR-T cells compared to 2028Z CAR-T cells (**Figure 2C**). Since 4-1BB signaling can help preserve the central memory phenotype of T cells (28, 31, 32), we also examined whether 4-1BB signaling affects the memory status of CAR-T in our newly designed construct. However, we did not observe significant differences in the proportion of central memory T cells (CCR7^+^CD45RO^+^CD45RA^-^) or effector memory T cells (CCR7^-^CD45RO^+^CD45RA^-^) between 2028Z-4-1BB CAR-T cells and 2028Z CAR-T cells during the culture period (**Figure 2D**).

### 4-1BB Co-Stimulation Signal Enhances the Survival of CAR-T Cells by Reducing Apoptosis

**Figure 3:**
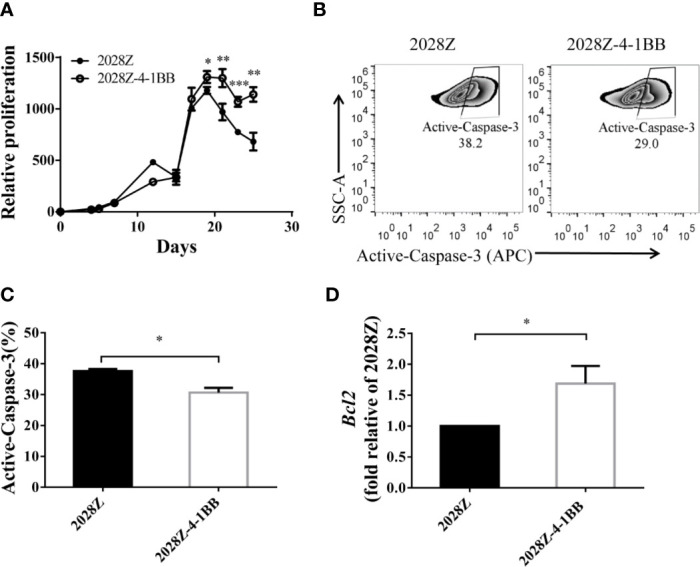
4-1BB signal improves *in vitro* proliferation of CAR-T cells. **(A)** Overall expansion of CAR-T cells in 2028Z and 2028Z-4-1BB CAR-T cell cultures. Relative cell proliferation was calculated by dividing the cell number by the cell number of Day 1. Experiments were repeated with three different donor-derived T cells (n = 3/group). Arrows indicate stimulation time points. **(B)** Apoptosis analysis of 2028Z and 2028Z-4-1BB CAR-T cells. Apoptosis was detected by active Caspase-3 staining. **(C)** Summarized active Caspase-3 staining data from three different donors. **(D)** Q-PCR analysis of the mRNA expression of *Bcl2* in 2028Z and 2028Z-4-1BB CAR-T cells. Representative results of one from three **(C, D)** repeated experiments are shown. *P < 0.05, **P < 0.01, ***P < 0.001.

An important aspect of CAR-T cell optimization is their enhanced proliferation and survival. Hence, we next compared the proliferation of 2028Z-4-1BB and 2028Z cells in our previously established *in vitro* long-term culture system (**Figure 2A**). The CAR^+^ cells in the 2028Z-4-1BB group showed significant survival enhancement compared with the 2028Z group, particularly in the later phase of long-term culture (**Figure 3A**). To evaluate whether reduced apoptosis contributed to enhanced survival, we compared the expression of active Caspase-3 in 2028Z-4-1BB and 2028Z CAR-T cells (**Figure 3B**) and observed a reduction in active Caspse-3 expression in 2028Z-4-1BB CAR-T cells (**Figure 3C**). Consistent with the phenotype of reduced apoptosis, expression of anti-apoptotic *Bcl2* was elevated in 2028Z-4-1BB CAR-T cells (**Figure 3D**). To test whether 4-1BB could enhance the CAR-T cell proliferation in the format of 3^rd^ generation or 4-1BB-incorporated 2^nd^ generation of CAR design, we established 2028BBZ and 20BBZ-4-1BB CAR-T cells (**Supplementary Figure 5A**). In 3^rd^ generation of CAR design, intracellular domain of 4-1BB was fused between CD28 and CD3ζ intracellular domain. In 4-1BB-incorporated 2^nd^ generation of CAR design, full length 4-1BB was linked to 20BBZ by a cleavable 2A peptide similarly as 2028Z-4-1BB. In long-term culture assay, 2028BBZ and 20BBZ-4-1BB CAR-T cells showed reduced proliferation ability compared with 2028Z CAR-T cells and 20BBZ CAR-T cells, respectively (**Supplementary Figures 5B, C**). These data collectively suggest that 4-1BB signaling can specifically enhance CAR-T cell survival through reducing apoptosis in CD28-incorporated 2^nd^ generation of CAR-T cells and we will focus on our study by dissecting the cytotoxic ability and *in vivo* efficacy of 2028Z-4-1BB CAR-T cells.

### 2028Z-4-1BB CAR-T Cells Are Effective Against Multiple Leukemia and Lymphoma *In Vitro*

**Figure 4:**
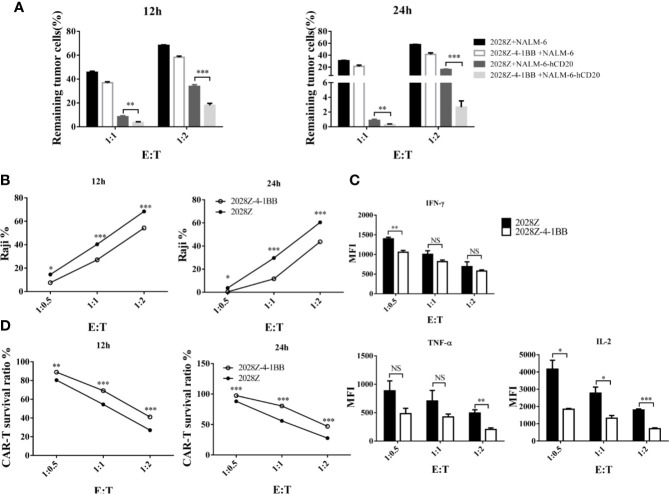
Profile of the cytotoxic ability of 2028Z-4-1BB CAR-T cells *in vitro*. **(A, B)** 2028Z and 2028Z-4-1BB CAR-T cells were co-cultured with NALM-6, NALM-6-hCD20 **(A)** or Raji **(B)** cells in triplicate at the different effector:target (E:T) ratios for 12-24 h. Relative cytotoxicity was calculated by analyzing the remaining tumor cells (CD3^-^CD19^+^) by flow cytometry. **(C)** 2028Z and 2028Z-4-1BB CAR-T cells were co-cultured with Raji cells for 12 h. Cytokines secreted by 2028Z and 2028Z-4-1BB CAR-T cells were determined by CBA. **(D)** 2028Z and 2028Z-4-1BB CAR-T cells were co-cultured Raji cells for 12–24 h. The relative survival of CAR-T cells was calculated by analyzing CD3^+^CD19^-^ cells by flow cytometry. Representative results of one from three **(A–D)** repeated experiments are shown. *P < 0.05, **P < 0.01, ***P < 0.001; NS: Not Significant.

Cytotoxicity ability is the most important feature of CAR-T cells, which directly determines their anti-tumor activity. To test whether 4-1BB signaling could enhance the cytotoxicity of CAR-T cells, we established an *in vitro* tumor killing assay in which CAR-T cells were co-cultured with various CD20^+^ tumor cells and tumor killing was determined by flow cytometry. First, we compared the cytotoxicity of 2028Z-4-1BB and 2028Z to that of the leukemia cell line NALM-6-hCD20 (**Supplementary Figure 6**). 2028Z-4-1BB CAR-T cells were observed to exhibit significantly higher killing capacity of leukemia cells compared to 2028Z CAR-T cells at E:T ratios of 1:1 and 1:2 (**Figure 4A**). We then tested whether this enhanced cytotoxicity could be applied to lymphoma cells. 2028Z-4-1BB CAR-T cells killed significantly more Raji lymphoma cells than 2028Z CAR-T cells (**Figure 4B**). These results suggest that 4-1BB enhance the cytotoxicity of CAR-T cells to a wide range of target cells. However, surprisingly, when the levels of specific cytotoxicity related effector molecules were assessed during the *in vitro* killing assay, 2028Z-4-1BB CAR-T cells were found to produce lower levels of TNF-α, IFN-γ, and IL-2 compared to 2028Z CAR-T cells (**Figure 4C**). We, therefore, further analyzed the survival of CAR-T cells and found that 2028Z-4-1BB CAR-T cells showed significantly higher survival ratios than 2028ZCAR-T cells (**Figure 4D**). These results suggested that the improved survival of 2028Z-4-1BB CAR-T cells may contribute to the enhanced cytotoxicity through a strategy of improving the quantity, not quality, of the CAR-T cells.

### 2028Z-4-1BB CAR-T Cells Show Better Persistence and Anti-Tumor Effects *In Vivo*

**Figure 5:**
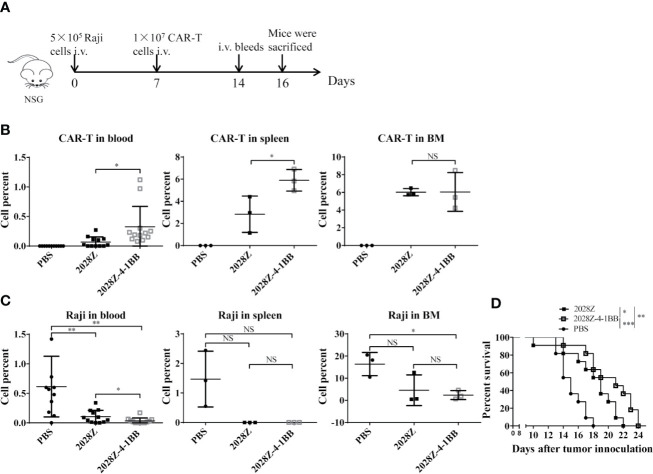
2028Z-4-1BB CAR-T cells show better persistence and anti-tumor effects *in vivo*. **(A)** A schematic diagram of the *in vivo* xenograft tumor model and CAR-T treatment protocol. **(B, C)** NSG mice were intravenously inoculated with 5x10^5^ Raji cells. Seven days later, tumor bearing mice were treated with 1x10^7^ 2028Z or 2028Z-4-1BB CAR-T cells. Nine days after the treatment, bone marrow, spleen (n = 3/group), and peripheral blood (n = 12/group) were collected for analysis of CAR-T cell (mCD45^-^hCD45^+^hCD3^+^) persistence and Raji (mCD45^-^hCD45^+^ hCD19^+^) tumor cell burden. **(D)** Kaplan-Meier analysis of the survival of mice (one point represents one mouse, n = 11/group). Representative results of one from three **(B–D)** repeated experiments are shown. *P < 0.05, **P < 0.01, ***P < 0.001; NS: Not Significant.

Our *in vitro* data indicated that 2028Z-4-1BB CAR-T cells exhibited enhanced proliferation and cytotoxicity, which prompted us to question whether these properties correlated with anti-tumor potency *in vivo*. To investigate this postulation, we establish a Raji lymphoma xenograft model in immunodeficient NOD/SCID/γ^−/−^ (NSG) mice for evaluating the therapeutic efficacy of 2028Z-4-1BB CAR-T cells (**Figure 5A**). We compared the persistence of CAR-T cells in peripheral blood, spleen, and bone marrow (**Figures 5B, C**). Significantly more CAR-T cells were observed in the 2028Z-4-1BB CAR-T cell-treated group compared to the 2028Z CAR-T cell-treated group in the peripheral blood and spleen. Consistent with the enhanced persistence of 2028Z-4-1BB CAR-T cells, the tumor burden in peripheral blood was lower in 2028Z-4-1BB CAR-T cell-treated mice. In the bone marrow, both the CAR-T cell persistence and tumor burden were similar. Moreover, both 2028Z and 2028Z-4-1BB CAR-T cell-treated groups exhibited significantly longer survival time compared to the PBS control group (**Figure 5D**). Due to their enhanced persistence and cytotoxicity *in vivo*, 2028Z-4-1BB CAR-T cell-treated Raji bearing mice showed a slightly prolonged overall survival compared to the 2028Z CAR-T cell treatment group (**Figure 5D**). Taken together, these results indicate that the 4-1BB co-stimulation signal enhanced the persistence of CAR-T cells *in vivo* and prolonged the survival of tumor bearing mice.

## Discussion

**Figure 6:**
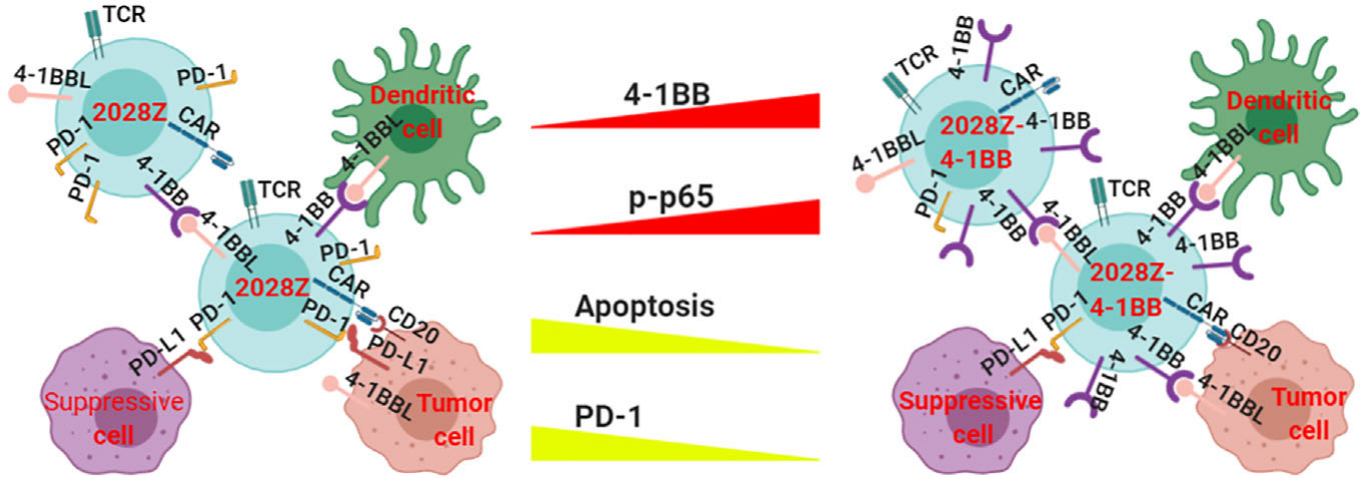
Proposed model of how 4-1BB signaling enhances the anti-tumor activity of 2028Z CAR-T cells. The NF-κB pathway is more active in 2028Z-4-1BB CAR-T cells than in 2028Z CAR-T cells. The expression of exhaustion marker PD-1 and pro-apoptotic active Caspase-3 is reduced in 2028Z-4-1BB CAR-T cells, compared with 2028Z CAR-T cells. These molecular mechanisms lead to enhanced proliferation, cytotoxicity ability, and anti-tumor activity of 2028Z-4-1BB CAR-T cells.

Although CAR-T cell immunotherapy has achieved remarkable efficacy for hematological malignancies (25, 33, 34), many challenges remain. The low persistence of CAR-T cells is one of the main obstacles in clinical treatment. Manipulation of the signal-chain variable fragment (scFv) (35), transmembrane domains (19), and co-stimulatory domains (27) is critical for enhancing CAR-T cell function and persistence. Among the co-stimulatory molecules incorporated into CARs, including OX40, ICOS, CD28 and 4-1BB, the most frequently used co-stimulatory domains are from CD28 and 4-1BB (36, 37). Second generation CARs containing CD28 or 4-1BB co-stimulatory molecules have shown different proliferation kinetics (26). The main characterization of CD28 based CAR-T cells is rapid expansion and poor persistence, which may limit their long-term efficacy. However, the 4-1BB co-stimulatory molecule confers CAR-T cells long-term expansion properties. To exploit the rapid expansion and circumvent the typical short persistence of CD28 based CAR-T cells, we co-expressed an intact co-stimulatory receptor 4-1BB to improve its persistence (**Figure 6**).

For the second generation CAR design, 4-1BB-incorporated CAR-T cells show milder activation and longer persistence than CD28-incorporated CAR-T cells, which leads to enhanced *in vivo* efficacy in preclinical tumor models (37–39). However, for the third generation CAR design, tandemly incorporated CD28 and 4-1BB co-stimulatory domains have shown controversial results in different studies (26, 27). In our study, we separately expressed an intact 4-1BB independent of the second generation CAR and observed enhanced persistence and cytotoxicity of 2028Z-4-1BB CAR-T cells compared with 2028Z CAR-T cells. However, 4-1BB didn’t promote CAR-T cell growth in the format of 3^rd^ generation of CAR design since the 2028BBZ CAR-T cells showed reduced proliferation compared with the 2028Z CAR-T cells. In clinical studies, the proliferation and persistence of CAR-T cells are important indicators for predicting efficacy of CAR-T therapy (40, 41). In our preclinical models, 2028Z-4-1BB CAR-T cells exhibited significantly enhanced proliferation and persistence compared with 2028Z CAR-T cells. Therefore, we hypothesized that 2028Z-4-1BB CAR-T cells are more effective in clinical settings. In addition, their improved proliferative capacity allows for reduced CAR-T cell therapeutic dosage, which will serve to reduce off-target side effects induced by CAR-T cell therapy (42), while also reducing the required time and cost associated with CAR-T cell manufacturing. When we applied the similar strategy to 4-1BB-incorporated 2^nd^ generation of CAR design, 20BBZ-4-1BB CAR-T cells didn’t show a proliferation benefit compared with 20BBZ CAR-T cells. This highlighted that 4-1BB can specifically enhance the proliferation ability of CD28-incorporated 2^nd^ generation of CAR-T Cells and it is important to explore other “synergistic co-stimulation pairs” for 3^rd^ generation or other 2^nd^ generation of CAR optimization.

Furthermore, this strategy raises the possibility that the activation style of the co-stimulatory domain is critical for modulating the function of CAR-T cells. When the co-stimulatory domain is integrated with the CAR design, it becomes activated simultaneously with CD3 by engagement of tumor specific antigen. Meanwhile, when a co-stimulatory receptor is independently expressed, it is activated by its natural ligand. Since co-stimulatory ligands are expressed by various types of cells and the expression can last for a certain period when the antigen is eliminated or down-regulated, they have the capacity to provide dynamic activation styles at different spatio-temporal dimensions (20, 21). Herein, we compared the mRNA expression level of 4-1BB ligand between tumor and adjacent normal tissues using the Tumor Immune Estimation Resource (TIMER, cistrome.shinyapps.io/timer). We found that various tumor tissues, including those of diffuse large B cell lymphoma (DLBCL), expressed higher levels of 4-1BB ligand mRNA (**Supplementary Figure 7**), suggesting that our novel 4-1BB expressing CAR-T cells may benefit from 4-1BB ligand enrichment in various tumor microenvironments. In addition, our novel CAR structure design provides a CAR development platform capable of mimicking the natural role of co-stimulatory signals during unmodified T cell activation. The various co-stimulatory receptors function differentially in the distinct T cell subsets and stages of T cell development (20, 21). It will, therefore, be interesting to assess the role of other co-stimulatory receptors in CAR-T functioning through our novel design strategy.

Several studies have demonstrated that 4-1BB promotes T cell activation and proliferation through triggering the activation of p38, JNK, and the NF-κB pathway (43–45). We have shown that activation of the NF-κB pathway was enhanced in 2028Z-4-1BB CAR-T cells, as indicated by the elevated expression of phospho-p65. A further systemic analysis of p38, JNK, and NF-κB pathway activation at different proliferation stages of 2028Z-4-1BB CAR-T cells will provide a more detailed activation mechanism of this novel CAR-T cell. Although the 4-1BB/4-1BBL signaling pathway can transduce the activation signal in both CD4^+^ and CD8^+^ T cells *in vitro*, it preferentially affects the development and activation of CD8^+^ T cells (43). In our study, we used total T cells for our CAR-T cell generation and observed an increase in the CD8^+^/CD4^+^ ratio in 2028Z-4-1BB CAR-T cells compared to 2028Z CAR-T cells, which is consistent with the natural role of 4-1BB in unmodified T cells. Interpretation of the differential role of 4-1BB signal in CD4^+^ and CD8^+^ CAR-T cells can be aided by using purified CD4^+^ or CD8^+^ T cells in the future. The critical immune checkpoint receptor PD-1 is significantly reduced in 2028Z-4-1BB CAR-T cells, suggesting a potential resistant mechanism in immune suppressive solid tumor microenvironment. In future studies, it will be interesting to design solid tumor targeting CAR-T with separated 4-1BB and test their anti-tumor efficacy in solid tumor model in further investigation.

CD19 is the most frequently used target for treating B cell derived leukemia and lymphoma. In our study, CD20 was selected as the target for CAR design as it is widely expressed at most stages of B cell development, and its expression occurs from pre-B cell to memory B cell stages (46). This unique expression of CD20 makes this marker an ideal target for CAR-T immunotherapy of B cell malignancies. In fact, clinical trials have demonstrated that the anti-CD20 antibody Rituximab has achieved remarkable efficacy for B cell malignancies with few side effects (47). Meanwhile, some patients undergo relapse due to loss of CD19 expression following CD19 targeted CAR-T therapy (48). Hence, our newly designed CD20 targeted CAR-T cells will provide a therapeutic option for CD19 negative patients. Furthermore, it will be interesting to investigate whether patients benefit from combinatorial CD19-targeting, and CD20-targeting CAR-T cell therapies.

In summary, we have developed a novel CAR construct with separated co-stimulatory receptor 4-1BB sensing. This strategy equipped CAR-T cells with distinct functional properties that mimic unmodified T cells, including enhanced proliferative capacity and effector functions compared to the widely used 28Z CAR-T cells. These characteristics led to enhanced anti-tumor efficacy in various B cell derived leukemia and lymphoma cells, both *in vitro* and *in vivo*. Our novel CAR design can be used in other CAR-T cells targeting different types of tumors, and it provides a new strategy for improving the efficacy of CAR-T cells.

## Data Availability Statement

All datasets generated for this study are included in the article/**Supplementary Material**.

## Ethics Statement

The animal study was reviewed and approved by Shanghai Jiao Tong University.

## Author Contributions

XY designed the overall project. QD, XZ, ML, XQ, HZ, PH, LF, FL, and XY performed the experiments. QD and XY analyzed the results and wrote the manuscript. All authors contributed to the article and approved the submitted version.

## Funding

XY was supported by the Program for Professor of Special Appointment (Eastern Scholar) at Shanghai Institutions of Higher Learning (TP2015013), The National Natural Science Foundation of China (81671643 and 81971467), The National Key Research and Development Program of China (2016YFC1303400), and the Recruitment Program of Global Experts (People’s Republic of China).

## Conflict of Interest

The authors declare that the research was conducted in the absence of any commercial or financial relationships that could be construed as a potential conflict of interest.
