# Supplementary material for: 4-1BB Signaling Boosts the Anti-Tumor Activity of CD28-Incorporated 2nd Generation Chimeric Antigen Receptor-Modified T Cells
Source: Front Immunol. 2020 Nov 13;11:539654. doi: 10.3389/fimmu.2020.539654 (PMC7691374; doi:10.3389/fimmu.2020.539654)
Supplement: Supplementary file 1 [file DataSheet_1.docx]

Supplementary Material

# Supplementary Figures


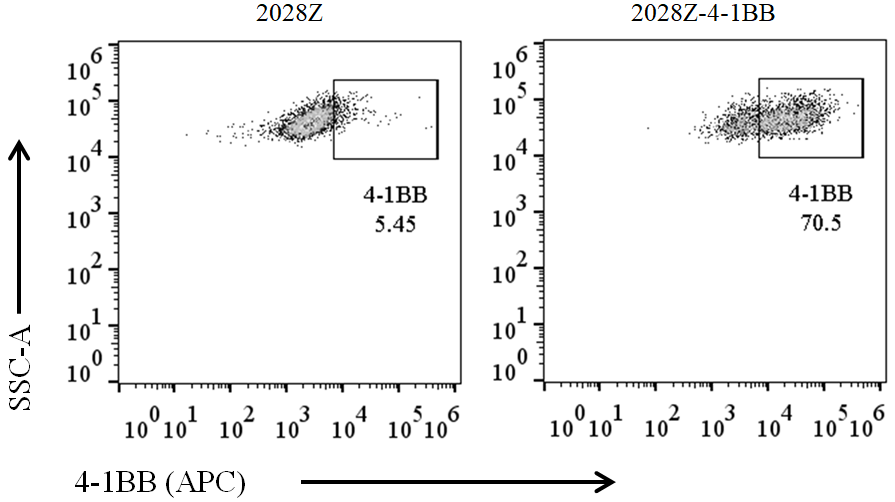


**Supplementary Figure 1.** Flow cytometry analysis of 4-1BB expression on 2028Z and 2028Z-4-1BB CAR-T cells.

**Supplementary Figure 2.** Q-PCR analysis of the mRNA levels of exogenous and endogenous 4-1BB in 2028Z and 2028Z-4-1BB CAR-T cells.


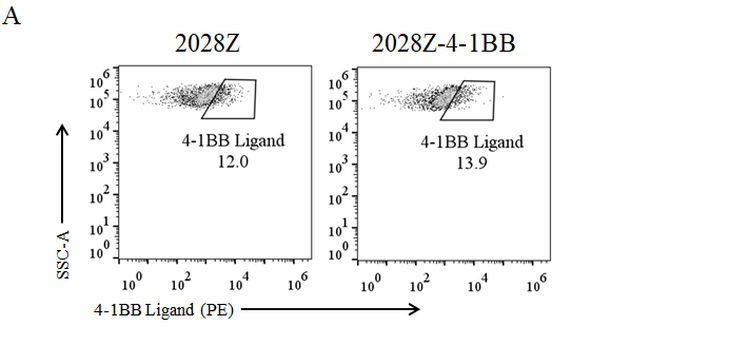


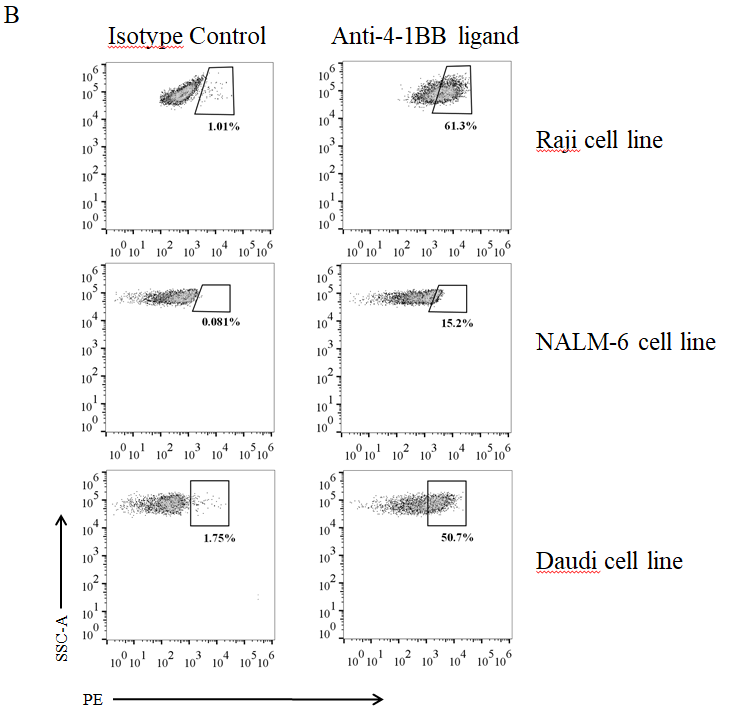


**Supplementary Figure 3.** Flow cytometry analysis of 4-1BB ligand expression on tumor cells or 2028Z and 2028Z-4-1BB CAR-T cells. A) Flow cytometry analysis of 4-1BB ligand expression on 2028Z and 2028Z-4-1BB CAR-T cells. B) Staining of Raji cells, NALM-6 cells and Daudi cells with mouse IgG1к isotype control or anti-human 4-1BB ligand PE.


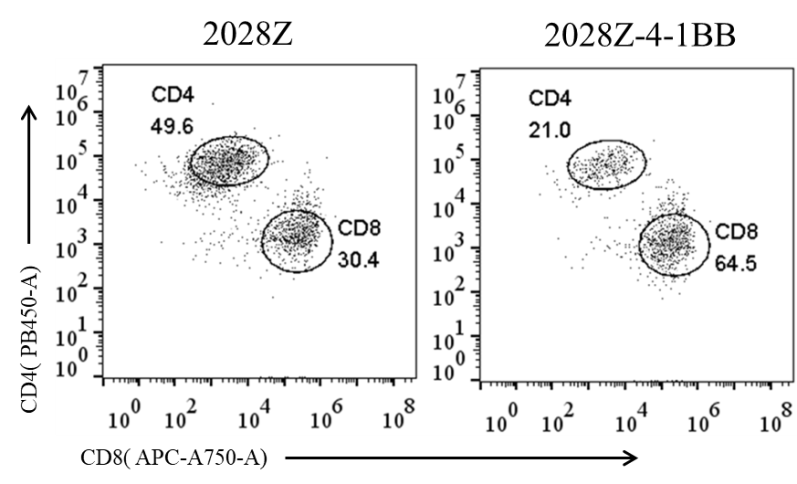


**Supplementary Figure 4.** Proportion of CD4^+^ and CD8^+^ CAR-T cells in the long-term culture as analyzed by flow cytometry.

A


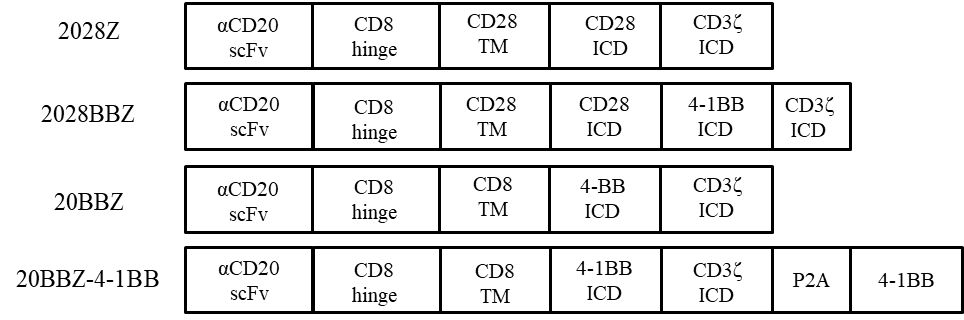


B

C

Supplementary Figure 5. Characterization of CAR-T cells with additional 4-1BB co-stimulatory molecule. A) A schematic diagram of 2028Z, 2028BBZ, 20BBZ and 20BBZ-4-1BB CAR-T cells. B-C) Overall expansion of CAR-T cells in 2028Z, 2028BBZ, 20BBZ and 20BBZ-4-1BB CAR-T cell cultures. Relative cell proliferation was calculated by dividing the cell number by the cell number of Day 0. Arrows indicate stimulation time points. Experiments were repeated with two different donor-derived T cells (n = 3/group).


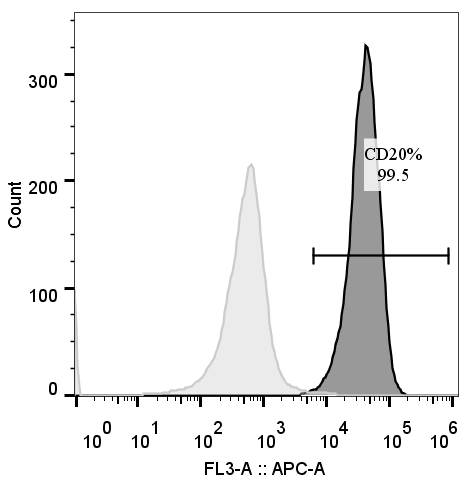


**Supplementary Figure 6.** Human CD20 expression on NALM-6 and NALM-6-hCD20 cell lines as determined by flow cytometry.


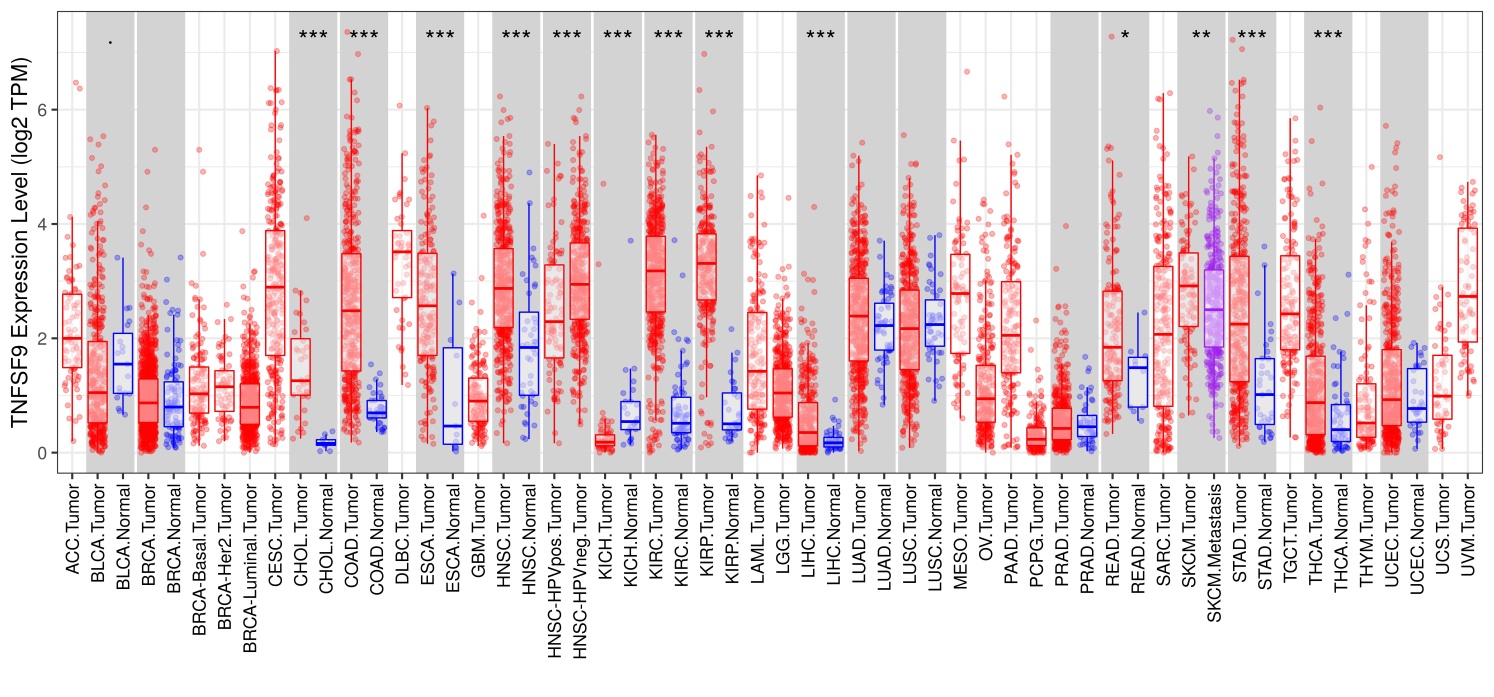
**Supplementary Figure 7.** 4-1BB ligand mRNA expression in different tumor types. Expression level of 4-1BB ligand (also referred to as TNFSF9) mRNA between tumor and adjacent normal tissues was analyzed using the Tumor Immune Estimation Resource (TIMER, cistrome.shinyapps.io/timer). Statistical analysis performed with the Wilcoxon test. P-value significance codes: 0 ≤ ******* < 0.001 ≤ ****** < 0.01 ≤ ***** < 0.05 ≤**•**< 0.1.
